# Data on the inhibition of RNase inhibitor activity by a monoclonal antibody as assessed by microfluidics-based RNA electrophoresis

**DOI:** 10.1016/j.dib.2016.09.010

**Published:** 2016-09-15

**Authors:** Xiao Wang, Belete Teferedegne, Kenneth Shatzkes, Wei Tu, Haruhiko Murata

**Affiliations:** Laboratory of DNA Viruses, Division of Viral Products, OVRR, CBER, FDA, Silver Spring, MD 20993, USA

**Keywords:** RNA, RNase, RNase inhibitor, Monoclonal antibody, Dithiothreitol

## Abstract

Using purified reaction components, a commercial monoclonal antibody (Ab) specific to RNase inhibitor (RI) was found to interfere with the activity of RI. Total RNA was mixed with a monoclonal Ab specific to either RI (clone 3F11) or glyceraldehyde-3-phosphate dehydrogenase (GAPDH), RNase A, RI, or a combination of the above. Following incubation for 1 h at 22 °C or 37 °C, RNA integrity of the mixtures was assessed using microfluidics-based Bio-Rad Experion RNA electrophoresis. The addition of Ab 3F11 prevented RI from effectively inhibiting RNase A and therefore resulted in extensive RNA degradation. The data presented are associated with the research article entitled “Endogenous RNase Inhibitor Contributes to Stability of RNA in Crude Cell Lysates: Applicability to Reverse Transcription Quantitative PCR (RT-qPCR)” (Wang et al., 2016) [1].

**Specifications Table**

**Table:** 

Subject area	*Biology*
More specific subject area	*Biochemistry, Molecular Biology*
Type of data	*Figures*
How data was acquired	*Microfluidics-based Bio-Rad Experion RNA electrophoresis*
Data format	*Raw*
Experimental factors	*RNA in solution was mixed with purified components (monoclonal antibodies, RNase A, human placental RNase inhibitor, dithiothreitol) and subjected to incubation at various temperatures*
Experimental features	*Following the incubation, RNA was purified and its integrity was assessed by Bio-Rad Experion RNA electrophoresis*
Data source location	*Food and Drug Administration (Silver Spring, Maryland, USA)*
Data accessibility	*Data is within this article*

**Value of the data**

• Using purified reaction components, a commercial monoclonal Ab specific to RI (3F11) was found to interfere with the activity of RI.
• This monoclonal Ab may be a useful reagent for exploring structure-function relationships of RI and for probing the interaction between RI and its target RNases.
• In addition, this monoclonal Ab may aid in establishing the role of RI as a determinant of RNA stability in complex mixtures (such as crude cell lysates).

## 1 Data

Mixtures of purified components (including total RNA, monoclonal Ab specific to RI or GAPDH, RNase A, RI, and dithiothreitol) were subjected to incubation for 1 h at various temperatures (on ice, 22 °C, or 37 °C). Following the incubation, RNA was purified from the mixtures and subjected to microfluidics-based Bio-Rad Experion RNA electrophoresis. Data shown are virtual gel images and RNA Quality Indicator (RQI) values generated by the Experion software.

The data presented are associated with the research article entitled "Endogenous RNase Inhibitor Contributes to Stability of RNA in Crude Cell Lysates: Applicability to RT-qPCR" [1].

## 2 Experimental design, materials and methods

### 2.1 Assessment of contaminating RNase activity in monoclonal Ab reagents

Total RNA from Vero cells was purified using the RNeasy kit (Qiagen) and diluted in Cell-Lysis (CL) Buffer (10 mM Tris–HCl pH 7.4, 0.25% Igepal CA-630, 150 mM NaCl) [2]. RNA (5 µg in 200 µL of CL Buffer) was mixed with 1 µg (1 µL) of monoclonal Ab specific to either RI (Origene; TA501875; clone 3F11) or GAPDH (Origene; TA802519; clone 2D9). The mixtures were incubated for 1 h on ice, at 22 °C, or at 37 °C. Following the incubation, RNA was purified using the RNeasy Mini kit (Qiagen) according to the “cleanup” protocol supplied with the kit. RNA was eluted in 30 µL of nuclease-free water and stored frozen at −80 °C until assessment. Samples (1 µL) were subjected to microfluidics-based Experion RNA StdSens electrophoresis (Bio-Rad). Virtual gel images and RQI values were generated using the Experion software version 3.2. RQIs can range from 1.0 for degraded RNA to 10.0 for intact RNA. According to the default Experion setting, RQIs between 7.0 and 10.0 indicate RNA of acceptable quality for most downstream applications such as RT-qPCR.

**Fig. 1:**
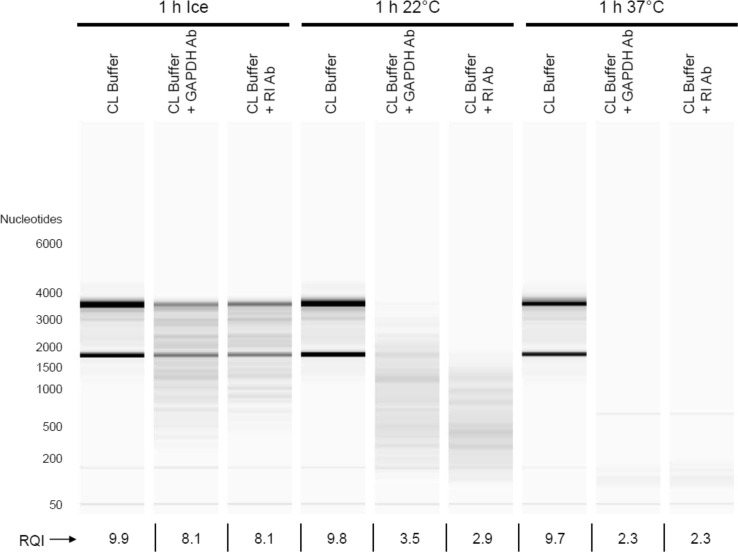
*Contaminating RNase activity in Ab reagents*. Purified total RNA (5 µg in 200 µL of CL Buffer) was mixed with 1 µg of monoclonal Ab specific to either RI (Origene; TA501875; clone 3F11) or GAPDH (Origene; TA802519; clone 2D9). The mixtures were incubated for 1 h on ice, at 22 °C, or at 37 °C. Following incubation, RNA was purified and subjected to Experion analysis.

Data from this experiment are shown in Fig. 1. The extent of RNase contamination was comparable between the two Ab reagents; according to information from the supplier, both were purified from mouse ascites fluid by affinity chromatography. RNA in control reactions in the absence of Ab (CL Buffer alone) was intact even after incubation for 1 h at 37 °C (RQI of 9.7), thereby demonstrating that our CL Buffer components are free of RNase activity.

### 2.2 Inhibition of RI activity by a monoclonal Ab specific to RI (3F11)

**Fig. 2:**
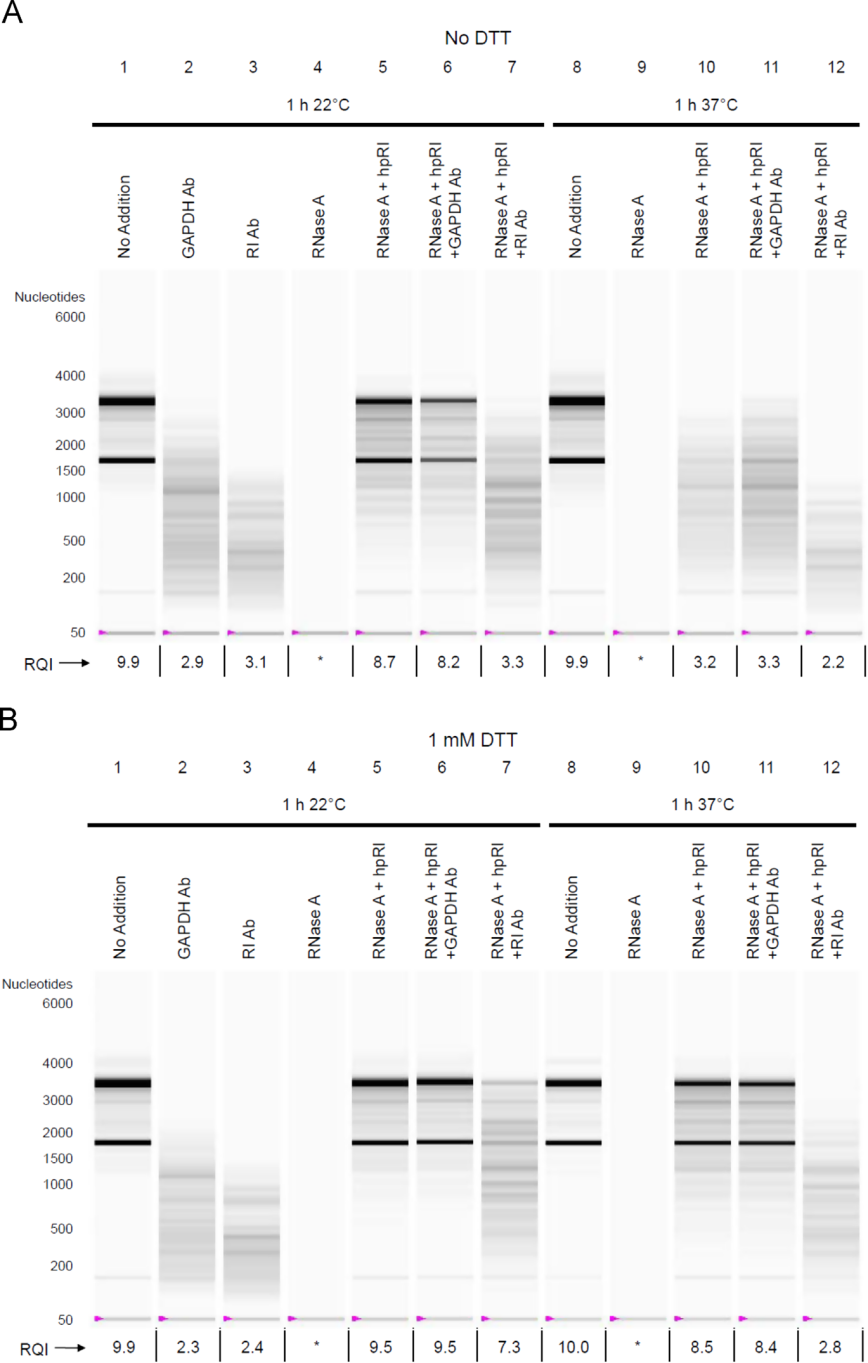
*Inhibition of RI activity by a monoclonal Ab specific to RI (3F11)*. (A) Purified total RNA (5 µg in 200 µL of CL Buffer) was mixed with 1 µg of monoclonal Ab specific to either RI (Origene; TA501875; clone 3F11) or GAPDH; (Origene; TA802519; clone 2D9), 1 ng of RNase A, 40 units of human placental RI (hpRI), or a combination of the above. The mixtures were incubated for 1 h at 22 °C or at 37 °C. Following incubation, RNA was purified and subjected to Experion analysis. An asterisk (^⁎^) indicates that the RNA concentration was too low for calculation of RQI. (B) A similar experiment as shown in (A) was performed using CL Buffer supplemented with 1 mM dithiothreitol (DTT).

Purified total RNA from Vero cells (5 µg in 200 µL of CL Buffer) was mixed with 1 µg (1 µL) of monoclonal Ab specific to either RI (Origene; TA501875; clone 3F11) or GAPDH (Origene; TA802519; clone 2D9), 1 ng (1 µL) of RNase A (Qiagen; 19101; diluted in CL Buffer), 40 units (1 µL) of human placental RI (hpRI; New England Biolabs; M0307), or a combination of the above; in reactions containing hpRI, its addition preceded the addition of other components. The mixtures were incubated for 1 h at 22 °C or at 37 °C. Following the incubation, RNA was purified and subjected to Experion analysis. Data from this experiment are shown in Fig. 2A. The nuclease activity associated with 1 ng of RNase A (lane 4) exceeded the contaminating nuclease activity associated with the monoclonal Ab reagents (lanes 2 and 3). The functionality of hpRI was demonstrated by protection against RNA degradation mediated by RNase A at 22 °C incubation for 1 h (lane 5); further addition of GAPDH Ab did not affect RNA stability (lane 6), whereas addition of RI Ab led to substantial RNA degradation (lane 7). At the more stringent stress condition (37 °C for 1 h), hpRI was not able to protect RNA from degradation (lanes 10 and 11), likely due to the instability of RI in the absence of reducing agent [3].

Fig. 2B represents data from a similar experiment using CL Buffer supplemented with 1 mM dithiothreitol (Thermo Scientific; 20291). RNA degradation mediated by RNase A was prevented by hpRI at both 22 °C (lane 5) and 37 °C (lane 10). Addition of RI Ab interfered with the function of hpRI at both 22 °C (lane 7) and 37 °C (lane 12), whereas addition of GAPDH Ab had minimal influence on RNA stability (lanes 6 and 11).
